# Exploiting Interdata Relationships in Prostate Cancer Proteomes: Clinical Significance of HO-1 Interactors

**DOI:** 10.3390/antiox11020290

**Published:** 2022-01-31

**Authors:** Sofia Lage-Vickers, Pablo Sanchis, Juan Bizzotto, Ayelen Toro, Agustina Sabater, Rosario Lavignolle, Nicolas Anselmino, Estefania Labanca, Alejandra Paez, Nora Navone, Maria P. Valacco, Javier Cotignola, Elba Vazquez, Geraldine Gueron

**Affiliations:** 1 Laboratorio de Inflamación y Cáncer, Departamento de Química Biológica, Facultad de Ciencias Exactas y Naturales, Universidad de Buenos Aires, Buenos Aires C1428EGA, Argentina; sofilage@qb.fcen.uba.ar (S.L.-V.); pablosanchis@qb.fcen.uba.ar (P.S.); jbizzotto@qb.fcen.uba.ar (J.B.); ayelentoro@qb.fcen.uba.ar (A.T.); asabater@qb.fcen.uba.ar (A.S.); rlavignolle@qb.fcen.uba.ar (R.L.); alejandravpaez@qb.fcen.uba.ar (A.P.); pvalacco@qb.fcen.uba.ar (M.P.V.); jcotignola@qb.fcen.uba.ar (J.C.); elba@qb.fcen.uba.ar (E.V.); 2 CONICET—Universidad de Buenos Aires, Instituto de Química Biológica de la Facultad de Ciencias Exactas y Naturales (IQUIBICEN), Buenos Aires C1428EGA, Argentina; 3 Department of Genitourinary Medical Oncology and the David H. Koch Center for Applied Research of Genitourinary Cancers, The University of Texas MD Anderson Cancer Center, Houston, TX 77030, USA; nanselmino@mdanderson.org (N.A.); elabanca@mdanderson.org (E.L.); nnavone@mdanderson.org (N.N.)

**Keywords:** *YWHAZ*, *HMOX1*, prostate cancer, proteomics, transcriptomics, protein interactions

## Abstract

Prostate cancer (PCa) cells display abnormal expression of proteins resulting in an augmented capacity to resist chemotherapy and colonize distant organs. We have previously shown the anti-tumoral role of heme oxygenase 1 (HO-1) in this disease. In this work, we undertook a mass spectrometry-based proteomics study to identify HO-1 molecular interactors that might collaborate with its modulatory function in PCa. Among the HO-1 interactors, we identified proteins with nuclear localization. Correlation analyses, using the PCa GSE70770 dataset, showed a significant and positive correlation between *HMOX1* and 6 of those genes. Alternatively, *HMOX1* and *YWHAZ* showed a negative correlation. Univariable analyses evidenced that high expression of *HNRNPA2B1, HSPB1, NPM1, DDB1, HMGA1, ZC3HAV1,* and *HMOX1* was associated with increased relapse-free survival (RFS) in PCa patients. Further, PCa patients with high *HSPB1/HMOX1*, *DDB1/HMOX1*, and *YWHAZ/HMOX1* showed a worse RFS compared with patients with lower ratios. Moreover, a decrease in RFS for patients with higher scores of this signature was observed using a prognostic risk score model. However, the only factor significantly associated with a higher risk of relapse was high *YWHAZ*. Multivariable analyses confirmed *HSPB1*, *DDB1*, and *YWHAZ* independence from PCa clinic-pathological parameters. In parallel, co-immunoprecipitation analysis in PCa cells ascertained HO-1/14-3-3ζ/δ (protein encoded by *YWHAZ*) interaction. Herein, we describe a novel protein interaction between HO-1 and 14-3-3ζ/δ in PCa and highlight these factors as potential therapeutic targets.

## 1. Introduction

Prostate cancer (PCa) is the second most common cancer type in men and the sixth leading cause of cancer-related death in men worldwide [1]. The discovery of new therapeutic avenues in PCa, and the development of effective drugs in the era of personalized medicine, would greatly benefit from the field of proteomics. The proteomics approach for a high-throughput study of biological samples by mass spectrometry has emerged as one of the main analytical strategies from the last years, and proteomic-based studies have greatly improved cancer research. Thus, proteomics represents an important tool for the identification of new molecular targets for PCa’s tailored therapy.

Inflammation is widely recognized as a hallmark of cancer [2]. Cell proliferation is enhanced in an inflammatory microenvironment rich in cytokines, growth factors, and agents that cause DNA damage [3]. This combination of factors makes the risk of developing a tumor much higher [3]. Further, inflammatory cells release reactive oxygen species (ROS), which generate oxidative stress and damage the DNA of neighboring epithelial cells, thus accelerating the evolution towards a more malignant phenotype [4]. Elevated intracellular ROS levels might affect several signaling pathways, resulting in the activation or repression of processes related to cell proliferation, motility, and survival [5,6]. Although the cause of prostatic inflammation is uncertain in most cases, it is believed that viral or bacterial infections, physical trauma from urinary reflux, dietary factors, estrogens, or a combination of these factors, could contribute to the establishment of an inflammatory microenvironment [7].

Heme oxygenase 1 (HO-1), the rate-limiting enzyme that catalyzes heme degradation, is a key player in cellular responses to pro-oxidative and pro-inflammatory insults [8,9]. HO-1 participates in cell homeostasis by attenuating inflammation, reducing oxidative injury, and regulating cell proliferation [10]. Reports from our laboratory documented that HO-1 has a strong anti-tumoral effect in vivo and in vitro in PCa [8,11,12,13,14,15,16]. Moreover, HO-1 induction in PCa cells impairs cell proliferation, invasion, and migration in vitro, and angiogenesis and tumor growth in vivo [11,12]. HO-1 is recognized as an integral smooth endoplasmic reticulum membrane protein; however, it has been detected in other subcellular compartments, including the nucleus [13,17,18]. It has been suggested that HO-1 undergoes proteolytic degradation at its carboxy-terminal hydrophobic end, which would facilitate its entry into the nucleus [13]. Interestingly, this truncated form of HO-1 does not possess catalytic activity [17]. It has been proposed that HO-1 has a non-canonical function in the nucleus, participating in the regulation of the activity of nuclear transcription factors and even regulating its own expression [19]. In line with this, we have previously documented that HO-1 binds to the proximal promoter of genes involved in PCa, such as the prostate-specific antigen (PSA), and represses androgen receptor (AR) activation revealing an undescribed function for HO-1 in the nucleus [12].

Due to the pleiotropic actions of HO-1, we hypothesized that its multiple functions could be mediated by interactions with several other relevant proteins associated with the carcinogenic process. Through co-immunoprecipitation assays, we previously verified that HO-1 interacts with STAT3, producing its retention in the cytoplasm of PCa cells [13]. Another HO-1 interactor protein in PCa cells identified by our group was Annexin A2 (ANXA2) [20], a key molecule in the adhesion process of PCa cells to the bone microenvironment. We found that HO-1 modulation in tumor cells interferes with ANXA2-mediated signaling [20]. These results clearly suggest that HO-1 is involved in cellular processes beyond the degradation of the heme group. However, further research into the mechanisms associated with HO-1 non-canonical functions is needed. Given that HO-1 does not show DNA binding motifs, it is possible that HO-1 needs to interact with transcription factors to fulfill its regulatory function in the cell nuclei.

To further our analysis, in this work, we undertook a proteomics approach to assess whether in PCa cells and under oxidative stress conditions, HO-1 could interact with proteins previously documented to have nuclear localization. Further, we evaluated the clinical relevance of such a network in PCa patients and performed correlation analyses among HO-1 and its partners, selecting those with higher correlation and building a risk score model. Taking into account all of our results, we report novel interactions between HO-1 and HSPB1, DDB1, and 14-3-3ζ/δ, highlighting their clinical relevance in PCa.

## 2. Materials and Methods

### 2.1. Cell Culture, Treatments, Reagents, and Antibodies

PC3 cells were obtained from the American Type Culture Collection (Manassas, VA, USA) and were routinely cultured in RPMI 1640 (Invitrogen, Grand Island, NY, USA) supplemented with 10% fetal bovine serum (FBS, Internegocios, Mercedes, Buenos Aires, Argentina), penicillin 100 U/mL, streptomycin 100 μg/mL, and amphotericin 0.5 μg/mL. Cells were cultured at 37 °C and 5% CO_2_.

For PC3 H_2_O_2_ treatment, cells were treated with 200 µM for 30 min, prepared in sterile PBS 1X. After treatment, cells were incubated in complete medium for 24 h and were harvested for the different experiments performed.

### 2.2. Antibodies

Monoclonal rabbit anti-human HO-1, anti-human 14-3-3ζ/δ and anti-human IgG antibodies were obtained from Cell Signaling (Danvers, MA, USA). Monoclonal mouse anti-human HO-1 antibody was obtained from Abcam (Cambridge, UK). Horseradish peroxidase (HRP) conjugated secondary anti-mouse antibody was obtained from Cell Signaling (Danvers, MA, USA). Secondary antibodies associated with the Alexa 555 and Alexa 647 fluorophores were obtained from Molecular Probes, Invitrogen (Carlsbad, CA, USA).

### 2.3. PEBG-GST-HO-1 Cloning

The vector pEBG-GST-HO-1 was generated by cloning the copy DNA sequence (cDNA) encoding the human HO-1 gene (*HMOX1*) into the restriction sites *BamHI* and *NotI* of the mammalian expression vector pEBG-GST (Addgene, Watertown, MA, USA). This strategy results in the fusion of the GST peptide at the N-terminus of HO-1. The sequences of primers used were: forward—5′-GCCGGATCCATGGAGCGTCCGCAAC-3′; reverse—5′-GCCGCGGCCGCCATTCACATGGCATAAAGC-3′.

### 2.4. Transfection with PEBG-GST and PEBG-GST-HO1

PC3 cells were transiently transfected with the HO-1 expression plasmid (pEBG-GST-HO-1) or the empty vector as control (pEBG-GST). Each 10 cm diameter plate was transfected using 10 μg of plasmid and 20 μL of polyethylene glycol (PEI) (Sigma-Aldrich, Gillingham, UK) in a final volume of 200 μL of RPMI 1640 culture medium. Transfections were performed on plates with 3 mL of RPMI without FBS or antibiotics. After 5 h of transfection, the culture medium was replaced by complete RPMI with 10% *v*/*v* FBS and antibiotics at the previously mentioned concentrations. For the proteomics tests, 40 plates of 10 cm in diameter of each experimental condition (GST-HO-1 vs. GST) were transfected and incubated for 48 h to carry out the different experiments.

### 2.5. GST Immunoprecipitation Strategy

48 h after transfection with pEBG-GST-HO-1 or the empty vector pEBG-GST and subsequent treatment with H_2_O_2_, proteins were extracted using a low concentration NaCl buffer (20 mM Tris, 150 mM NaCl, 5 mM MgCl2, 0.5% NP40, pH 7.5) to avoid disruption of protein-protein interactions. Protein extracts were incubated for 2 h at 4 °C with beads coated with glutathione-S-agarose.

### 2.6. Separation of Peptides and Mass Spectrometry Analysis

Recombinant GST-HO-1 protein complexes were reduced (200 mM DTT), alkylated (200 mM iodoacetamide), and digested with trypsin in-solution overnight, using an estimated 1:30 enzyme to substrate ratio. The peptides were desalted and concentrated in a C18 resin (Zip-Tips, Waters Technologies Corporation, Milford, MA, USA) before analysis by LC ESI-MS/MS at the Center for Metabolomics and Mass Spectrometry (The Scripps Research Institute, La Jolla, CA, USA). Peptides were separated by reverse-phase chromatography before mass spectrometry analysis using the following method: nanoelectrospray capillary column tips were made in-house by using a P-100 laser puller (Sutter Instruments, Novato, CA, USA). The columns were packed with Zorbax SB-C18 stationary phase (Agilent Technologies, Santa Clara, CA, USA) purchased in bulk (5 mm particles, with a 15 cm length and a 75 mm inner diameter). The reverse-phase gradient separation was performed by using water and acetonitrile (0.1% formic acid) as the mobile phases. The gradient consisted of 5% acetonitrile for 10 min followed by a gradient to 8% acetonitrile for 5 min, 35% acetonitrile for 113 min, 55% acetonitrile for 12 min, 95% acetonitrile for 15 min, and re-equilibrated with 5% acetonitrile for 15 min.

Data-dependent MS/MS data were obtained with an LTQ linear ion trap mass spectrometer using a home-built nanoelectrospray source at 2 kV at the tip. One MS spectrum was followed by 4 MS/MS scans on the most abundant ions after the application of a dynamic exclusion list. Tandem mass spectra were extracted using the Xcalibur software (Thermo Scientific, Waltham, MA, USA). All MS/MS samples were analyzed by using Mascot (version 2.1.04; Matrix Science, London, UK) with *H. Sapiens* proteins contained in the NCBI protein database (NCBInr2 20080628 (6655203 sequences; 2281585098 residues). Taxonomy: *Homo sapiens* (human) (202147 sequences), assuming the digestion enzyme trypsin with a maximum of 1 miscleavage. Mascot was searched with a fragment ion mass tolerance of 0.80 Da and a parent ion tolerance of 2.0 Da, and fixed modifications: carbamidomethyl (C) and variable modifications: oxidation (M). Identification was carried out at the 95% confidence level with a calculated false-positive rate of <1% as determined by using a reversed concatenated protein database. The minimum score for a nonrandom identification with more than 95% confidence was 45 for the GST control search and 44 for the GST-HO-1 search. At least one unique peptide with MS/MS data was identified for each protein hit. To control for nonspecific binding, we compared GST-HO-1 co-purifying proteins with those immunoprecipitated in cells transfected with a GST empty vector. Only differential GST-HO-1 binding proteins compared with GST-binding proteins were considered further.

### 2.7. Co-Immunoprecipitation (Co-IP)

After treatment with H_2_O_2_, total proteins were extracted from PC3 cells and quantified using the BCA method (Sigma-Aldrich, Gillingham, UK) (98% BCA + 2% CuSO_4_). 500 µg of proteins from the cell lysates and 10 µg of the specific rabbit anti-human HO-1 antibody were diluted in a final volume of 500 µL in RIPA buffer (150 µM NaCl, 20 µM EDTA 1% *v*/*v* sodium deoxycholate, 0.1% *v*/*v* sodium dodecyl sulfate (SDS), 1% Triton x-100Tris, pH 7.4). As a specificity control, samples were incubated with 10 µg of nonspecific rabbit anti-human IgG antibody. All samples were supplemented with MPI protease inhibitors (Sigma-Aldrich, Gillingham, UK), and were incubated overnight at 4 °C with orbital shaking. Protein A/G PLUS-Agarose beads (Santa Cruz Biotechnology Inc., Santa Cruz, CA, USA) were washed; 20 µL were added to each sample and incubated overnight at 4 °C with orbital shaking. Antibody-protein-beads complexes were washed with 500 μL of RIPA buffer and re-suspended in 40 μL of RIPA 20% loading buffer (5% B-Mercaptoethanol). Samples were heated for 5 min at 95 °C. 60 µg of the total protein lysate was used as input. The immune complexes were analyzed by Western blot as previously described [21], using mouse anti-human HO-1 or rabbit anti-human 14-3-3ζ/δ antibodies. Three independent experiments were performed.

### 2.8. Immunofluorescence (IF) Experiment

60,000 PC3 cells were cultured on coverslips placed in 12-well plates and the respective treatments were carried out. Afterwards, cells were washed three times with PBS and then fixed with 1 mL of 100% methanol for 20 min at −20 °C. After removing the methanol, cells were incubated at room temperature in a permeabilizing solution of 1 mL PBS-triton 0.1% *v*/*v* for 10 min. Subsequently, coverslips were placed in a humid and dark chamber and were incubated with a blocking solution (40 μL of PBS-BSA 3% m/v for 1 h). Coverslips were then incubated overnight at 4 °C with 40 µL of the corresponding primary antibodies (mouse anti-human HO-1 and/or rabbit anti-human 14-3-3ζ/δ antibodies), prepared in PBS-BSA 3% m/v. After performing three washes of 10 min with PBS, the coverslips were incubated for 1 h at room temperature with the Alexa Fluor 555 and/or Alexa Fluor 647 secondary antibodies in a 1:5000 dilution prepared in PBS-BSA 3% m/v.

After three washes of 10 min with PBS, the coverslips were mounted on a slide with 2 µL of Mowiol (Sigma-Aldrich, Gillingham, UK) and stored at 4 °C in the dark until use.

Confocal images were acquired by confocal microscopy (FV1000, Olympus, Tokyo, Japan), using a UPlanSApo 100X oil immersion objective (NA 1/41.35; Olympus), a diode laser of 543 and 635 nm as the excitation sources. Images were obtained with a Qimaging EXI Aqua camera; >20 cells were analyzed for the IF assay for each condition.

### 2.9. Image Processing for Presentation

Confocal images were processed for presentation using ImageJ software (NIH, Bethesda, MD, USA). The background of each channel was subtracted. The JACoP plugin was used in order to evaluate co-localization, and Manders and Pearson’s coefficients were estimated. GraphPad Prism software (La Jolla, CA, USA) was used to assess statistical significance, which was set at *p* < 0.05.

### 2.10. Bioinformatics Analysis

#### 2.10.1. Identification of HO-1 Interactor Proteins with Nuclear Localization and GO Enrichment Analysis

Protein–protein interactions and gene ontology (GO) analyses were performed using the STRING webtool [22], using a minimum combined interaction score of 0.400. Plots were generated using Cytoscape [23] and enrichplot package [24] in R. Statistical significance was set at false discovery rate (FDR) *p* < 0.05.

#### 2.10.2. Information Source and Eligibility Criteria (GEO: Gene Expression Omnibus)

To study the impact of the expression of the selected genes on the survival of patients, we selected the following dataset:

Ross-Adams 2015 (GSE70770) GPL10558 series [25]: a PCa patient’s cohort with 206 tumor tissue samples from men with PCa who had undergone radical prostatectomy and a clinical follow-up of 9 years, including biochemical relapse (BCR) information, defined according to European Guidelines as a persistent rise of PSA above 0.2 ng/mL. Tumor sample expression of 31,000 transcripts was measured by 47,000 probes using the Illumina Human HT-12 V4.0 platform. A descriptive table regarding patient characteristics at baseline (start of the follow-up for survival analyses) is depicted in Supplementary Table S1.

#### 2.10.3. Gene Correlation

Pairwise gene correlation between *HMOX1* and all genes encoding for the HO-1 interactor proteins of interest was analyzed with the ggcorrplot package in R. Correlation coefficients were classified as weak (|r| ≤ 0.30), intermediate (0.30 < |r| < 0.66), and strong (|r| ≥ 0.66). Statistical significance was set at *p* < 0.05.

#### 2.10.4. Risk Scoring System Analysis

Based on the expression of genes with a strong/intermediate gene correlation with *HMOX1*, a risk score model was created based on the coefficients of a Cox logistic regression analysis. Then, the sum of the product of the coefficient (Coef) values of all genes and their dichotomic expression (Expr) values was calculated as the patient risk score (risk score = $\overset{n}{\underset{i=1}{\sum }}\left(Coef_{i}\;\times \;Expr_{i}\right)$). Using Cutoff Finder software [26], patients were divided into high-risk and low-risk groups. Kaplan–Meier survival analysis was used to determine whether relapse-free survival (RFS) was significantly different between high-risk and low-risk patients.

#### 2.10.5. Survival Analyses

Kaplan–Meier curves showing the RFS of patients with PCa were plotted using the survminer package [27] in R. To find the optimal cutoff value to stratify patients into two groups based on the expression levels of each gene, we used the Cutoff Finder tool. Multivariable analysis was performed in Stata (StataCorp LLC, College Station, TX, USA) and plotted in GraphPad Prism software (La Jolla, CA, USA). For univariable and multivariable analyses of prognostic factors, the Log-rank test and Cox proportional hazard model regression were employed. Statistical significance was set at *p* < 0.05.

## 3. Results

### 3.1. Proteomics Profile of HO-1 Interactors in PCa Cells

We have previously identified HO-1 molecular partners involved in cell–cell communication and cell adhesion through an integrative “omics” approach [28], establishing four molecular pathways (ANXA2/HMGA1/POU3F1; PLAT/PLAU; TMOD3/RAI14/VWF; NFRSF13/GSN). Further, we also described ANXA2, a protein associated with both bone physiology and in PCa bone progression [29,30], as an HO-1 interactor [20].

**Figure 1:**
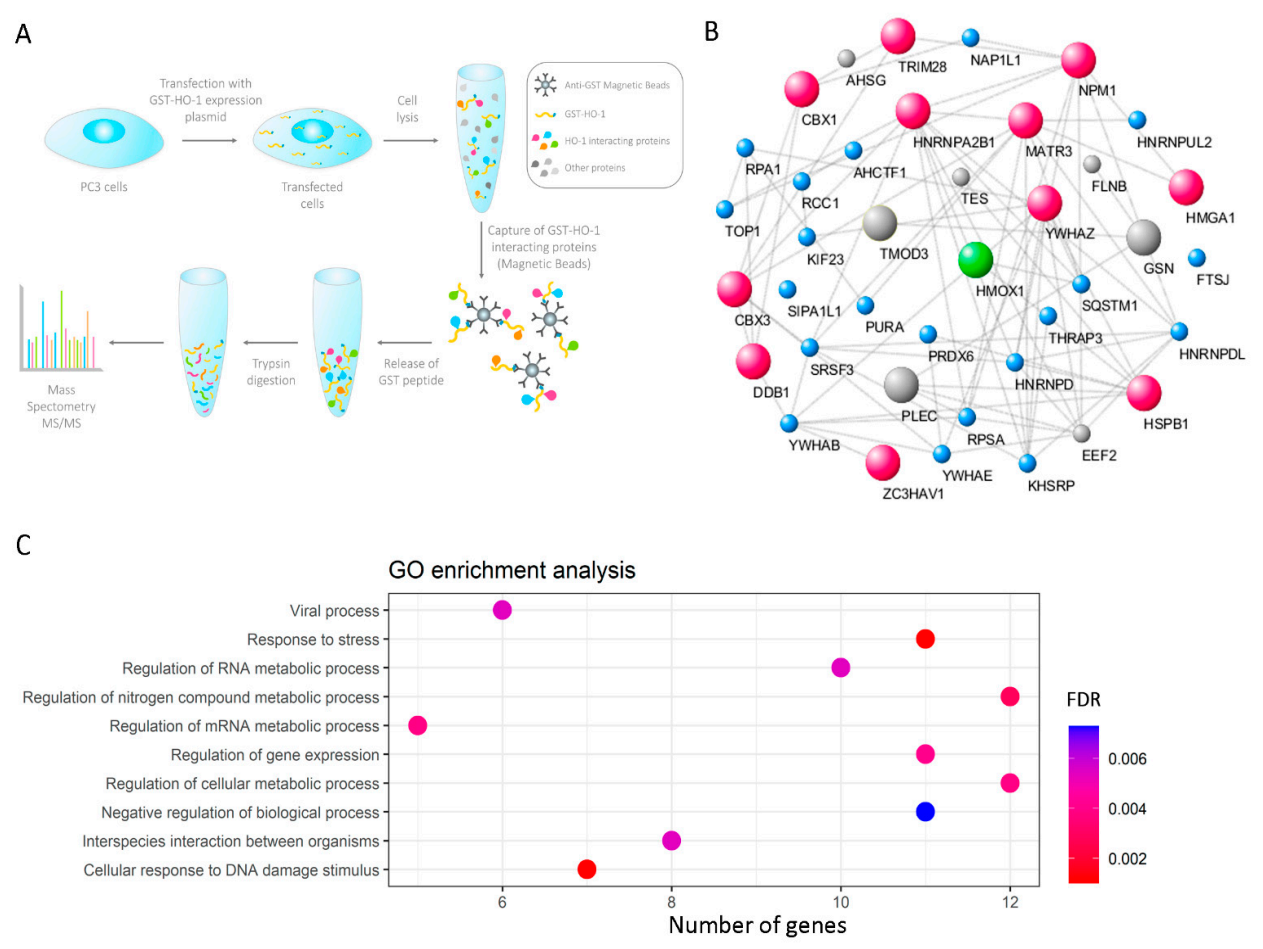
Construction of the HO-1 interactome in PCa cells. (**A**) Simplified schematic workflow of the construction of the HO-1 interactome in PC3 cells. GST-HO-1 immunoprecipitation assays were performed from PC3 cell extracts that had been previously treated with H_2_O_2_ (200 μM, 1 h). For the LC ESI-MS/MS analysis, peptides were desalted and concentrated on a C18 resin. Tandem mass spectra were extracted using the Xcalibur software. All MS/MS samples were analyzed by using Mascot. (**B**) Interactions network for the GST-HO-1 binding proteins. The network was built with Cytoscape 3.7.0. using the interactions data obtained with STRING, using a minimum combined interaction score of 0.400. The protein size increases proportionally to the PSMs obtained in the LC ESI-MS/MS analysis. Proteins in pink are proteins identified with PSM > 2 and previously reported in the cell nucleus. (**C**) Gene ontology (GO) (biological processes) categories significantly dysregulated for genes encoding for HO-1 interactor proteins with nuclear localization. The color gradient shows the adjusted *p*-value. FDR = false discovery rate.

In light of the strong anti-tumoral role of HO-1 in PCa, we deepened our analyses in the search for other HO-1 interactors with clinical relevance for the disease. We carried out a proteomics analysis, in which PC3 cells, derived from a bone metastasis of PCa [31], were transfected with the HO-1 expression plasmid (pEBG-GST-HO-1) or with the empty vector as control (pEBG-GST) (Figure 1A). After 48 h, cells were treated with 200 μM H_2_O_2_ for 30 min, with the aim of generating an inflammatory and oxidative microenvironment similar to the one which characterizes PCa [7]. Subsequently, GST-HO-1 was immunoprecipitated together with its interactors. The eluates were digested for analysis by LC ESI-MS/MS (Figure 1A). We identified 41 HO-1 interactor proteins, including GSN (Gelsolin), FLNB (filamin B), 14-3-3 family proteins, TES (testin), TRIM28 (tripartite motif-containing 28), and SRSF3 (splicing factor rich in serine and arginine 3), with clinical relevance in PCa [32,33,34,35,36,37]. Supplementary Table S2 shows the differential proteins identified (GST-HO-1 vs. GST) together with the number of unique peptides, the name of the encoding gene, the score, and the percentage of coverage with respect to the complete sequence. Next, an interaction network was built using STRING [22] and Cytoscape [23], which is shown in Figure 1B. Significant protein hits (Supplementary Table S2) identified with more than 2 PSMs were selected for further analysis (Figure 1B, larger spheres). This filter resulted in the selection of 14 proteins. Among those, 11 proteins had been previously reported with nuclear localization, including tripartite motif-containing 28 (TRIM28), heterogeneous nuclear ribonucleoprotein A2/B1 (HNRNPA2B1), heat shock protein family B (small) member 1 (HSPB1), chromobox 1 (CBX1), chromobox 3 (CBX3), matrin 3 (MATR3), nucleophosmin 1 (NPM1), damage specific DNA binding protein 1 (DDB1), high mobility group AT-hook 1 (HMGA1), 14-3-3 zeta delta-like protein (14-3-3ζ/δ) and zinc finger CCCH-type containing, antiviral 1 (ZC3HAV1) [22] (Figure 1B, pink spheres). The gene ontology (GO) for the top biological processes (BP) categories of the genes encoding for the HO-1 interactor proteins with nuclear localization included response to stress and cellular response to DNA damage stimulus (Figure 1C).

### 3.2. Gene Correlation between HMOX1 and the Genes Encoding for HO-1 Interactors with Nuclear Localization in PCa Cells

**Figure 2:**
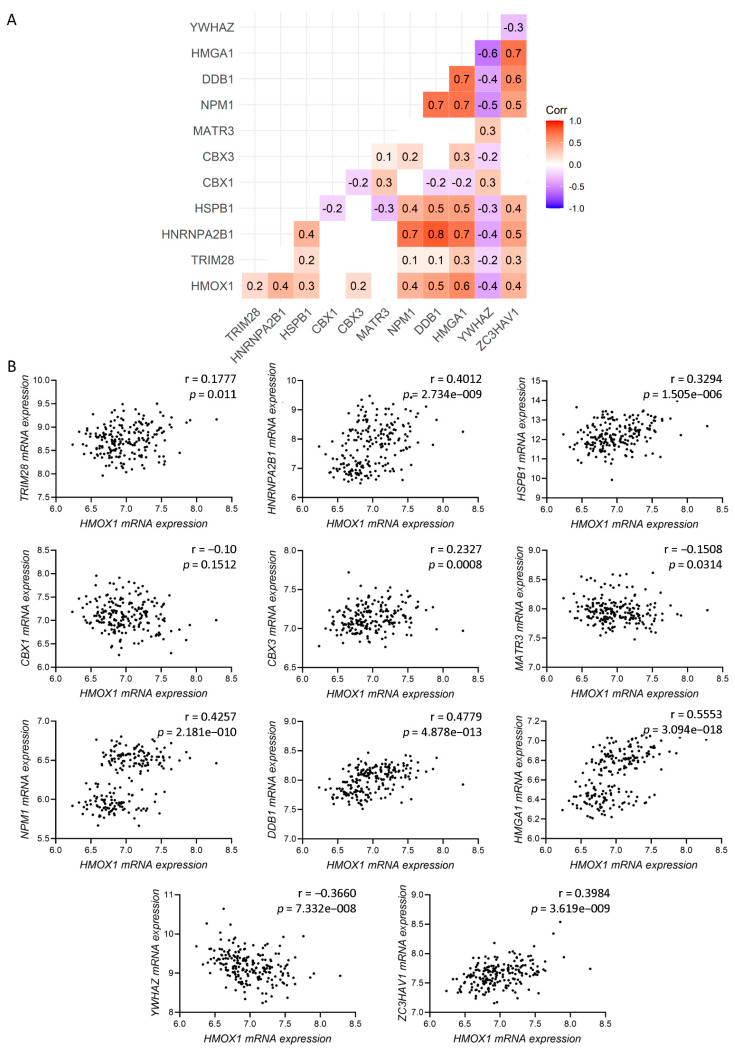
Gene correlation. (**A**) Pairwise Spearman correlation matrix analysis between *HMOX1* and all of the genes encoding for the HO-1 interactor proteins which were previously reported in the cell nucleus (*TRIM28*, *HNRNPA2B1*, *HSPB1*, *CBX1, CBX3*, *MATR3*, *NPM1*, *DDB1*, *HMGA1*, *YWHAZ*, and *ZC3HAV1*). Rounded Spearman correlation values are included inside each color box. Color scale ranges from blue (r = −1) to white (r = 0) to red (r = 1). (**B**) Spearman correlation analysis for *HMOX1* and the genes encoding for the HO-1 interactor proteins, which were previously reported in the cell nucleus (*TRIM28*, *HNRNPA2B1*, *HSPB1*, *CBX1*, *CBX3*, *MATR3*, *NPM1*, *DDB1*, *HMGA1*, *YWHAZ*, and *ZC3HAV1*). Statistical significance was set at *p* < 0.05.

To study the relevance of the HO-1 interactors in PCa, we assessed the clinical significance of these nuclear factors by using the GSE70770 dataset [25], which gathers transcriptomic and clinical data from PCa patients who had undergone radical prostatectomy and clinical follow-up of 9 years, including biochemical relapse. Next, we performed pairwise correlation analyses between *HMOX1* and the genes encoding for the 11 selected nuclear localized HO-1 interactors (Figure 2B). Results show a significant and positive intermediate/strong Spearman correlation between *HMOX1* and 6 of those genes (r = 0.4, *p* < 0.0001 for *HNRNPA2B1*; r = 0.3, *p* < 0.0001 for *HSPB1*; r = 0.4, *p* < 0.0001 for *NPM1*; r = 0.5, *p* < 0.0001 for *DDB1*; r = 0.6, *p* < 0.0001 for *HMGA1*; and r = 0.4, *p* < 0.0001 for *ZC3HAV1*). Interestingly, *HMOX1* only correlated negatively with *YWHAZ* (r = −0.4, *p* < 0.0001) (Figure 2A,B).

### 3.3. Clinical Relevance of HO-1 Interactors with Nuclear Localization in PCa

**Figure 3:**
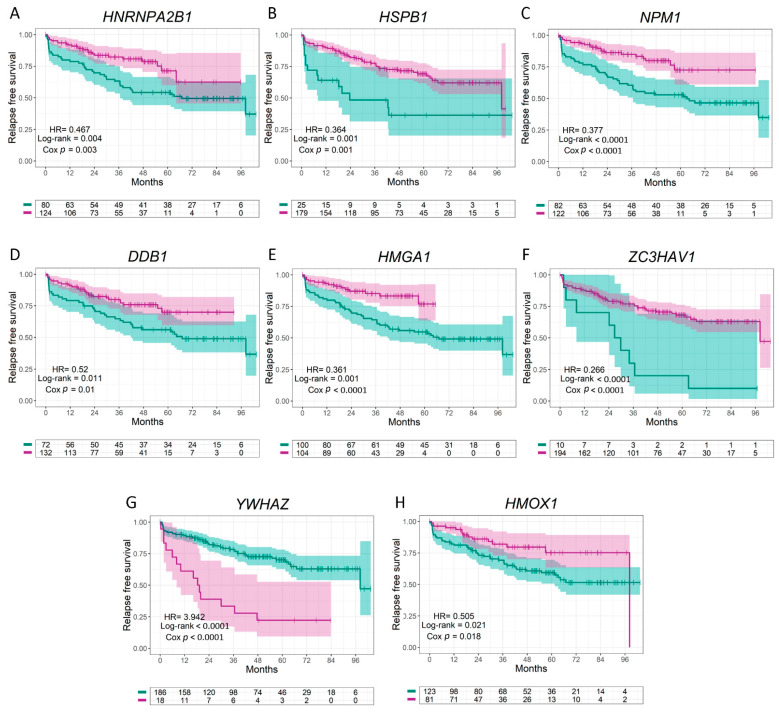
Relapse-free survival (RFS) of PCa patients using the Ross–Adams dataset, GSE70770, n = 206. (**A**–**H**) Kaplan–Meier curves for RFS of PCa patients segregated based on the gene expression levels for *HNRNPA2B1* (**A**), *HSPB1* (**B**), *NPM1* (**C**), *DDB1* (**D**), *HMGA1* (**E**), *ZC3HAV1* (**F**), *YWHAZ* (**G**), and *HMOX1* (**H**). RFS of patients with high (purple lines) vs. low (green lines) expression for each gene. HR = hazard ratios (95% confidence interval). All comparisons consider low expression patients as the reference group. Cox *p* = Cox proportional hazard model *p*-value. Statistical significance was set at Cox *p* < 0.05.

In order to evaluate the clinical relevance of the HO-1 interactors that have been previously reported in the nucleus, we analyzed the biochemical relapse-free survival (RFS) of PCa patients associated with the expression of the genes encoding for those proteins. The analyzed genes were the ones which showed a significant (*p* < 0.05) and intermediate (0.30 < |r| < 0.66) or strong (|r| ≥ 0.66) mRNA correlation with *HMOX1* (*HNRNPA2B1, HSPB1, NPM1, DDB1, HMGA1, ZC3HAV1,* and *YWHAZ*). We also included *HMOX1* expression in this analysis. We plotted KM curves for each individual gene assessed (Figure 3). Patients were stratified into two groups based on the expression levels for each gene: high and low expression. The analysis showed that high expression of *HNRNPA2B1*, *HSPB1*, *NPM1*, *DDB1*, *HMGA1*, *ZC3HAV1*, and *HMOX1* was associated with an increased RFS in PCa patients (HR = 0.467, Cox *p* = 0.003 for *HNRNPA2B1* (Figure 3A); HR = 0.364, Cox *p* = 0.001 for *HSPB1* (Figure 3B); HR = 0.377, Cox *p* < 0.0001 for *NPM1* (Figure 3C); HR = 0.52, Cox *p* = 0.01 for *DDB1* (Figure 3D); HR = 0.361, Cox *p* < 0.0001 for *HMGA1* (Figure 3E); HR = 0.266, Cox *p* < 0.0001 for *ZC3HAV1* (Figure 3F); and HR = 0.505, Cox *p* = 0.018 for *HMOX1* (Figure 3H)). However, high expression of *YWHAZ* was associated with a lower RFS (HR = 3.942, Cox *p* < 0.0001) (Figure 3G).

**Figure 4:**
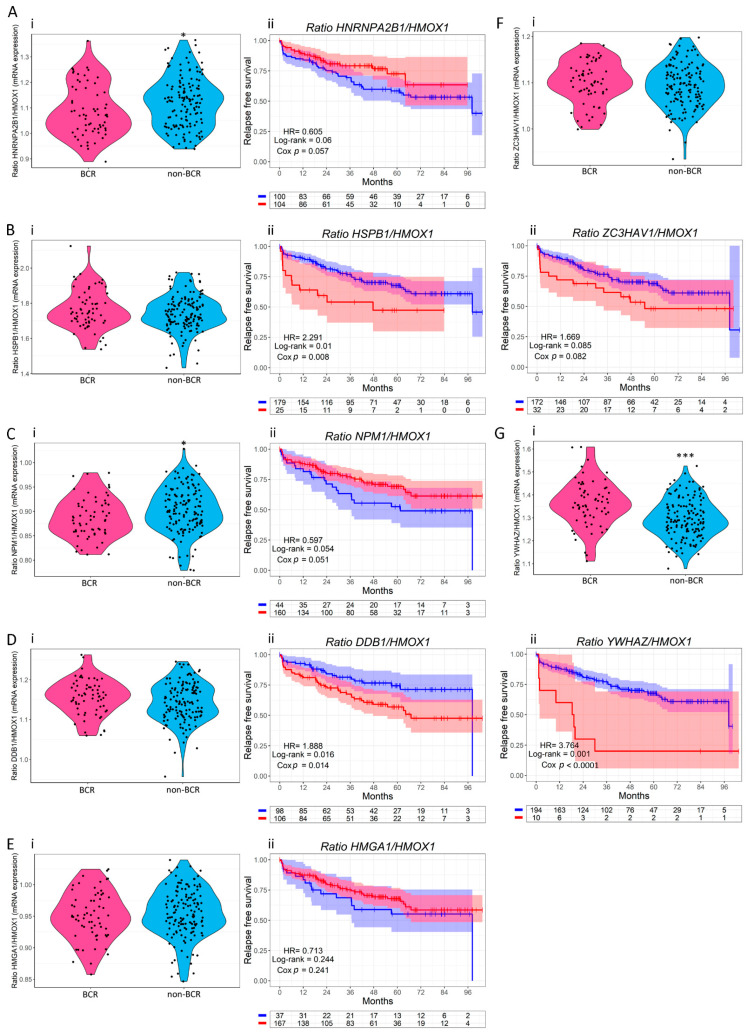
Expression ratios between *HNRNPA2B1*, *HSPB1*, *NPM1*, *DDB1*, *HMGA1*, *ZC3HAV1*, *YWHAZ*, and *HMOX1* and their association with biochemical relapse (BCR) and relapse-free survival (RFS) using the Ross-Adams dataset, GSE70770, n = 206. (**i**) Violin plots depicting *HNRNPA2B1/HMOX1* (**A**), *HSPB1/HMOX1* (**B**), *NPM1/HMOX1* (**C**), *DDB1/HMOX1* (**D**), *HMGA1/HMOX1* (**E**), *ZC3HAV1/HMOX1* (**F**), and *YWHAZ/HMOX1* (**G**) expressions in BCR vs. non-BCR patients. (**ii**) Kaplan–Meier curves for RFS of PCa patients segregated based on the gene expression *HNRNPA2B1/HMOX1* (**A**), *HSPB1/HMOX1* (**B**), *NPM1/HMOX1* (**C**), *DDB1/HMOX1* (**D**), *HMGA1/HMOX1* (**E**), *ZC3HAV1/HMOX1* (**F**), and *YWHAZ/HMOX1* (**G**) ratios. RFS of patients with high (red lines) vs. low (blue lines) expression for each ratio. HR = hazard ratios [95% confidence interval]. All comparisons consider low expression patients as the reference group. Cox *p* = Cox proportional hazard model *p*-value. Statistical significance was set at Cox *p* < 0.05. * *p* < 0.05; *** *p* < 0.001.

Next, we furthered our analysis by plotting the gene expression ratio between each of these seven genes and *HMOX1* in biochemical relapse (BCR) patients compared with non-BCR. Results show that *HNRNPA2B1/HMOX1*, *NPM1/HMOX1*, and *YWHAZ/HMOX1* were significantly higher in BCR compared with non-BCR patients (*p* = 0.028 for *HNRNPA2B1/HMOX1* (Figure 4(Ai)); *p* = 0.018 for *NPM1/HMOX1* (Figure 4(Ci)); *p* < 0.0001 for *YWHAZ/HMOX1* (Figure 4(Gi))). Next, we plotted the RFS stratifying patients according to their gene expression ratio. PCa patients with higher *HSPB1/HMOX1*, *DDB1/HMOX1*, and *YWHAZ*/*HMOX1* showed a worse RFS compared with patients with lower ratios (HR = 2.291, Cox *p* = 0.006 for *HSPB1/HMOX1* (Figure 4(Bii)), HR = 1.888, Cox *p* = 0.014 for *DDB1/HMOX1* (Figure 4(Dii)), and HR = 3.764, Cox *p* < 0.0001 for *YWHAZ*/*HMOX1* (Figure 4(Gii))). Results evidenced that increased *HMOX1* expression in combination with high expressions of *HSPB1*, *DDB1*, and *YWHAZ*, improves RFS for PCa patients.

**Figure 5:**
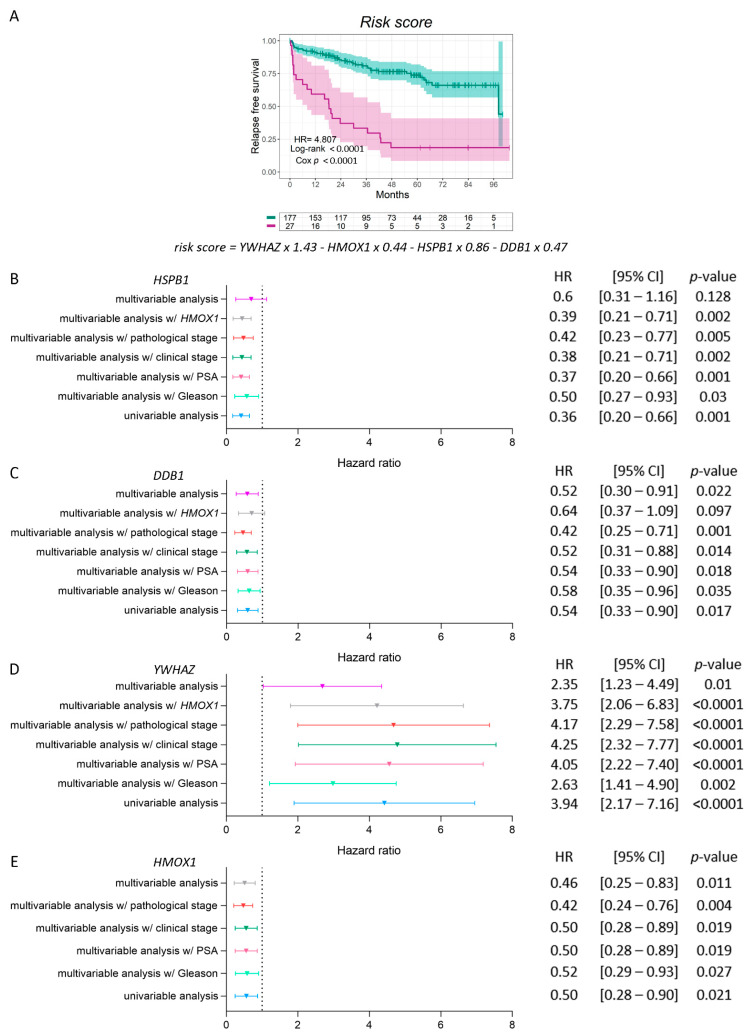
Risk score regression model and multivariable analyses for *HSPB1*, *DDB1*, *YWHAZ**,*** and *HMOX1* in PCa patients. (**A**) Kaplan–Meier curve for RFS in high-risk (purple lines) and low-risk (green lines) groups, according to a risk score model based on the expression of *HSPB1*, *DDB1*, *YWHAZ*, and *HMOX1* in PCa patients. (**B**–**D**) Multivariable analyses presented by forest plots between each gene (*HSPB1* (**B**), *DDB1* (**C**), *YWHAZ* (**D**), and *HMOX1* (**E**)) and GS, PSA, clinical and pathological stage, *HMOX1*′s expression, or all the variables together. Univariable analysis (light blue); multivariable analysis with GS (light green) = adjusted for the GS (6; 7 (3 + 4); 7 (4 + 3); 8-10); multivariable analysis with PSA (pink) = adjusted for the PSA serum levels at diagnosis (PSA (ng/mL): <4; 4-10; > 10); multivariable analysis with the clinical stage (dark green) = adjusted for the clinical stage; multivariable analysis with the pathological stage (red) = adjusted for the pathological stage; multivariable analysis with *HMOX1* (grey) = adjusted for the expression of *HMOX1*; multivariable analysis (purple) = adjusted for all the variables simultaneously. HR = hazard ratios (95% confidence interval). All comparisons consider low expression patients as the reference group. Cox *p* = Cox proportional hazard model *p*-value. Statistical significance was set at Cox *p* < 0.05.

We then performed a Cox regression analysis and built a prognostic model for predicting the RFS, based on the expression (Expr) and coefficient (Coef) values of *HMOX1* and each of the three genes whose higher ratios with *HMOX1* showed a significant decrease in RFS compared with patients with lower ratios (*YWHAZ*, *DDB1*, and *HSPB1*) (Table 1). The risk score was calculated as follows: =$\overset{n}{\underset{i=1}{\sum }}\left(Coef_{i}\;\times \;Expr_{i}\right).\;$ On the basis of the result for each PCa patient, the GSE70770 dataset was divided into two groups (high-risk group and low-risk group) according to the optimal cutoff value. Interestingly, we observed that patients with higher risk scores had a worse clinical outcome than patients with lower risk scores (HR = 4.807, Cox *p* < 0.0001) (Figure 5A).

To validate *HSPB1*, *DDB1*, *YWHAZ*, and *HMOX1*’s clinical relevance in PCa, multivariable analyses were performed in the presence of clinic-pathological parameters previously associated with increased PCa relapse risk. These parameters included Gleason score (GS), PSA levels, patients’ clinical and pathological stages, and *HMOX1* expression. *HSPB1* behaved independently from the patients’ GS, PSA levels, clinical and pathological stages, and *HMOX1* expression (*p* = 0.03 for GS; *p* = 0.001 for PSA; *p* = 0.002 for the clinical stage; *p* = 0.005 for the pathological stage; *p* = 0.002 for *HMOX1* (Figure 5B)). *DDB1* behaved independently from the patients’ GS, PSA levels, and clinical and pathological stages (*p* = 0.035 for GS; *p* = 0.018 for PSA; *p* = 0.014 for the clinical stage; and *p* = 0.001 for the pathological stage (Figure 5C)). When we further adjusted the model to include all variables simultaneously, the associations remained significant (*p* = 0.022) (Figure 5C). Further, *YWHAZ* behaved independently from the patients’ GS, PSA levels, clinical and pathological stages, and *HMOX1* expression (Figure 5D). When analyzing all variables simultaneously, the associations remained significant (*p* = 0.01) (Figure 5D). Figure 5E depicts *HMOX1* independence from all of the clinic-pathological parameters previously analyzed (*p* = 0.027 for GS; *p* = 0.019 for PSA; *p* = 0.019 for the clinical stage; *p* = 0.004 for the pathological stage; *p* = 0.023 for all the variables simultaneously (Figure 5E)). Altogether, the multivariable analyzes add support to the independence of variables to predict the patient outcome.

### 3.4. Validation of the Interaction between HO-1 and 14-3-3ζ/δ in PCa Cells

Considering that: (1) *YWHAZ* has been reported to be an independent and strong predictor of aggressiveness in PCa [38]; (2) its expression showed a significant negative correlation with *HMOX1* expression; (3) PCa patients with high *YWHAZ/HMOX1* showed the highest HR; and (4) high *YWHAZ* was the only factor significantly associated with a higher risk of relapse; we validated the interaction between HO-1 and 14-3-3ζ/δ. PC3 cells were treated with H_2_O_2_ or vehicle, and protein eluates were subjected to co-immunoprecipitation. As seen in Supplementary Figure S1A, HO-1 and 14-3-3ζ/δ interact in PC3 cells.

In addition, by confocal microscopy, HO-1 and 14-3-3ζ/δ co-localization was evaluated in PC3 cells treated with H_2_O_2_ or vehicle. The images displayed in Supplementary Figure S1B showed that HO-1 and 14-3-3ζ/δ co-localize in the cell nucleus under the induction of HO-1 with H_2_O_2_ (Supplementary Figure S1B). When analyzing the Manders and Pearson co-localization coefficients, a significant increase is observed in PC3 cells treated with H_2_O_2_ compared with controls (Supplementary Figure S1C).

Altogether, we confirmed for the first time the interaction between HO-1 and 14-3-3ζ/δ, highlighting them as critical players in PCa, and potential targets for clinical intervention.

## 4. Discussion

HO-1 is a key player in the cellular defense system against pro-oxidative and pro-inflammatory insults [10]. Regarding its role in pathological conditions, this protein is commonly considered as a survival molecule that plays an important role in cancer [10]. However, there is controversy about its role in tumor development and progression, possibly because its expression profile is associated with the type of tissue in question and depends, in turn, on the context or the tumor microenvironment.

Previous reports from our laboratory documented for the first time that HO-1 is expressed in human primary prostate carcinomas and is localized in the cell nucleus [18]. In PCa cell lines, we found that the pharmacological and genetic induction of HO-1 inhibits proliferation, migration, and invasion in vitro; further, it slows down tumor growth, limits tumor-associated angiogenesis [12] and neovascularization [15], and boosts the antitumor response in vivo [15].

Given the pleiotropic anti-tumoral role of HO-1 in PCa, in this work, we set out to evaluate whether HO-1 interacted with proteins previously described with nuclear localization, enabling it to reprogram prostate tumor cells fate, favoring the acquisition of a less aggressive phenotype. After generating oxidative stress conditions, HO-1 co-immunoprecipitates were subjected to LC ESI-MS/MS, identifying 11 proteins reported with nuclear localization. Interestingly, GO analyses showcased response to stress and cellular response to DNA damage stimulus as the top significant biological processes categories, highlighting the non-canonical HO-1 potential nuclear function in PCa.

Our next aim was to evaluate the clinical relevance of such interactors in association with HO-1 in PCa patients that had undergone radical prostatectomy (GSE70770) [25]. Gene expression correlation analyses showed a significant and positive Spearman correlation between *HMOX1* and *HNRNPA2B1*, *HSPB1*, *NPM1*, *DDB1*, *HMGA1*, and *ZC3HAV1*. Of note, *HMOX1* and *YWHAZ* showed a significant negative correlation.

We next set out to study whether the ratios of *HNRNPA2B1*/*HMOX1*, *HSPB1*/*HMOX1*, *NPM1*/*HMOX1*, *DDB1*/*HMOX1*, *HMGA1*/*HMOX1*, *ZC3HAV1*/*HMOX1*, and *YWHAZ*/*HMOX1* might affect patients RFS. Results confirmed that PCa patients with higher *HSPB1*/*HMOX1*, *DDB1*/*HMOX1*, and *YWHAZ*/*HMOX1* showed a worse RFS, highlighting the protective role of HO-1.

Further, we computed a risk score constituted by *HSPB1*, *DDB1*, *YWHAZ*, and *HMOX1,* evidencing a decrease in the RFS of patients with higher risk scores. Multivariable analyses supported the independence of variables to predict the patient outcome.

Interestingly, it has been reported that HSPB1 correlates with the overall survival of patients with several types of cancer. Particularly, HSPB1 induction is associated with highly aggressive disease and poor clinical outcomes in PCa. At early tumor stages, HSPB1 expression is inhibited, but it is re-expressed during PCa progression, leading to a more aggressive phenotype [39,40]. On the other hand, Zoubeidi et al. described a novel cooperative interaction between AR and HSPB1 that enhances AR stability and transcriptional activity, thereby increasing prostate cancer cell survival [41].

DDB1 is involved in DNA repairing and has been related to tumor suppression [42,43]. Regarding its role as a member of the CUL4A-DDB1 E3 ligase complex, it promotes ubiquitination-dependent AR degradation. Accordingly, DDB1 and AR protein levels negatively correlate in PCa cells [44]. 14-3-3ζ/δ is an adapter protein encoded by *YWHAZ*. Members of the 14-3-3 protein family are involved in the regulation of a wide spectrum of signaling pathways by binding to various proteins [45] and contributing to the regulation of crucial cellular processes such as protein trafficking, malignant transformation, and differentiation [46,47]. Particularly, the ζ/δ isoform constitutes a potential prognostic and therapeutic target since its high expression correlates with the progression of different cancers [38,48,49]. Previous studies from our group demonstrated *YWHAZ* relevance as a prognostic factor independent from the clinic-pathological parameters associated with the disease, such as age, GS, and TMPRSS2-ERG fusion [38]. Moreover, we observed that PCa patients with amplification, or increased mRNA or protein levels for *YWHAZ*, have significant alterations in key DNA repair genes [38].

In terms of PCa immunotherapy, different therapeutic mechanisms have been described for these 3 HO-1 interactors: HSPB1, DDB1, and 14-3-3ζ/δ. It has been reported a novel immune escape mechanism mediated by HSPB1, in which this protein expressed in the breast tumor microenvironment, promotes the differentiation of monocytes to macrophages with immune-tolerogenic phenotypes, which, in turn, trigger severe anergy in T-cells [50]. DDB1 has been proposed as a key factor for immunomodulatory drug sensitivity [51]. Moreover, DDB1 has been identified in high-throughput analyses as a potential target, showing sensitivity to Poly(ADP-ribose) polymerase (PARP) inhibition [52]. This therapeutic avenue might be combined together with different immunomodulatory drugs, enhancing their effect [53]. In the case of 14-3-3ζ/δ, Yu et al. [54] reported that immune-associated genes involved in interferon signaling, TLR-4 signaling, inflammasome network, antigen presentation/TCR recognition, and CD28 co-stimulation were found significantly downregulated in patients with urothelial carcinomas of the urinary bladder (UCUBs) presenting *YWHAZ* amplification/overexpression. However, there is no evidence of *YWHAZ* immunomodulation in PCa. Further studies are required in order to determine whether an anti-*YWHAZ* approach might be a useful strategy for improving the therapeutic efficacy of immunotherapy in PCa.

Remarkably, 14-3-3 proteins can affect various physical and functional aspects of their targets, such as: blocking nuclear localization or export signals affecting their subcellular localization, blocking their binding to other proteins, affecting their stability, and modulating their catalytic activity by modifying their conformation [47,55,56,57,58]. In most cases, the binding of 14-3-3 sequesters the target protein in a particular subcellular compartment, and the release of 14-3-3 allows the protein to relocate. Likewise, recently Chen et al. [58] revealed through in vitro and in vivo studies that 14-3-3ζ/δ promotes invasion and metastasis of non-small-cell lung cancer by binding to the soluble form of β-catenin phosphorylated at Ser552, protecting it from ubiquitin-mediated degradation, suggesting a novel mechanism by which β-catenin accumulates in the cytoplasm and remains protected from degradation in tumoral cells. Hence, 14-3-3ζ/δ promotes the activation of the Wnt pathway and the transcriptional activity of β-catenin, which enters the nucleus and interacts with the TCF/LEF complexes, inducing epithelial-mesenchymal transition, proliferation, and cell migration. Furthermore, it is widely accepted that the canonical Wnt pathway modulates osteoblast function and participates in the induction of the osteoblastic phenotype in PCa bone metastasis [59]. These results show the importance of the 14-3-3ζ/δ/β-catenin axis in PCa progression.

In this work, we confirmed the HO-1/14-3-3ζ/δ interaction, performing a co-immunoprecipitation assay. Further, through an immunofluorescence assay, we determined that HO-1 and 14-3-3ζ/δ co-localize in the nucleus of PCa cells under oxidative stress conditions. Remarkably, this is the first time that this interaction has been reported in this type of cell. Although Song et al. reported this interaction in hepatocellular carcinoma [55], they only observed co-localization of both proteins in the cell cytoplasm, demonstrating that the interaction inhibited the ubiquitination and subsequent degradation of HO-1, facilitating its stability.

## 5. Conclusions

In summary, the results obtained in this study describe for the first time the interaction between HO-1 and HSPB1, DDB1, and 14-3-3ζ/δ in PCa cells. Further work will be necessary to identify the fine molecular mechanisms tuning HO-1 towards the acquisition of a less aggressive phenotype and to delineate the role of its interactions in PCa.

## Figures and Tables

**Table 1:** Detailed information of HO-1 interactors for the risk score model.

Gene	Coeff.	[95% CI]	*p*-Value
*HMOX1*	−0.44	−1.04–0.16	0.147
*YWHAZ*	1.43	0.82–2.04	<0.001
*DDB1*	−0.48	−1.01–0.05	0.08
*HSPB1*	−0.86	−1.47–−0.25	0.006

## Data Availability

The data is contained in the manuscript and supplementary file.

## Supplementary Materials

The following supporting information can be downloaded at: https://www.mdpi.com/article/10.3390/antiox11020290/s1, Figure S1: Validation of the interaction between 14-3-3ζ/δ and HO-1 by co-immunoprecipitation and fluorescence microscopy; Table S1: Ross-Adams patients’ characteristics at baseline (start of the follow-up survival analyses); Table S2: Differential proteins identified by mass spectrometry in GST-HO-1 vs. GST.
