# Supplementary material for: Exploiting Interdata Relationships in Prostate Cancer Proteomes: Clinical Significance of HO-1 Interactors
Source: Antioxidants (Basel). 2022 Jan 31;11(2):290. doi: 10.3390/antiox11020290 (PMC8868058; doi:10.3390/antiox11020290)
Supplement: Supplementary file 1 [file antioxidants-11-00290-s001.zip › antioxidants-1520695-supplementary.pdf]

Supplementary information

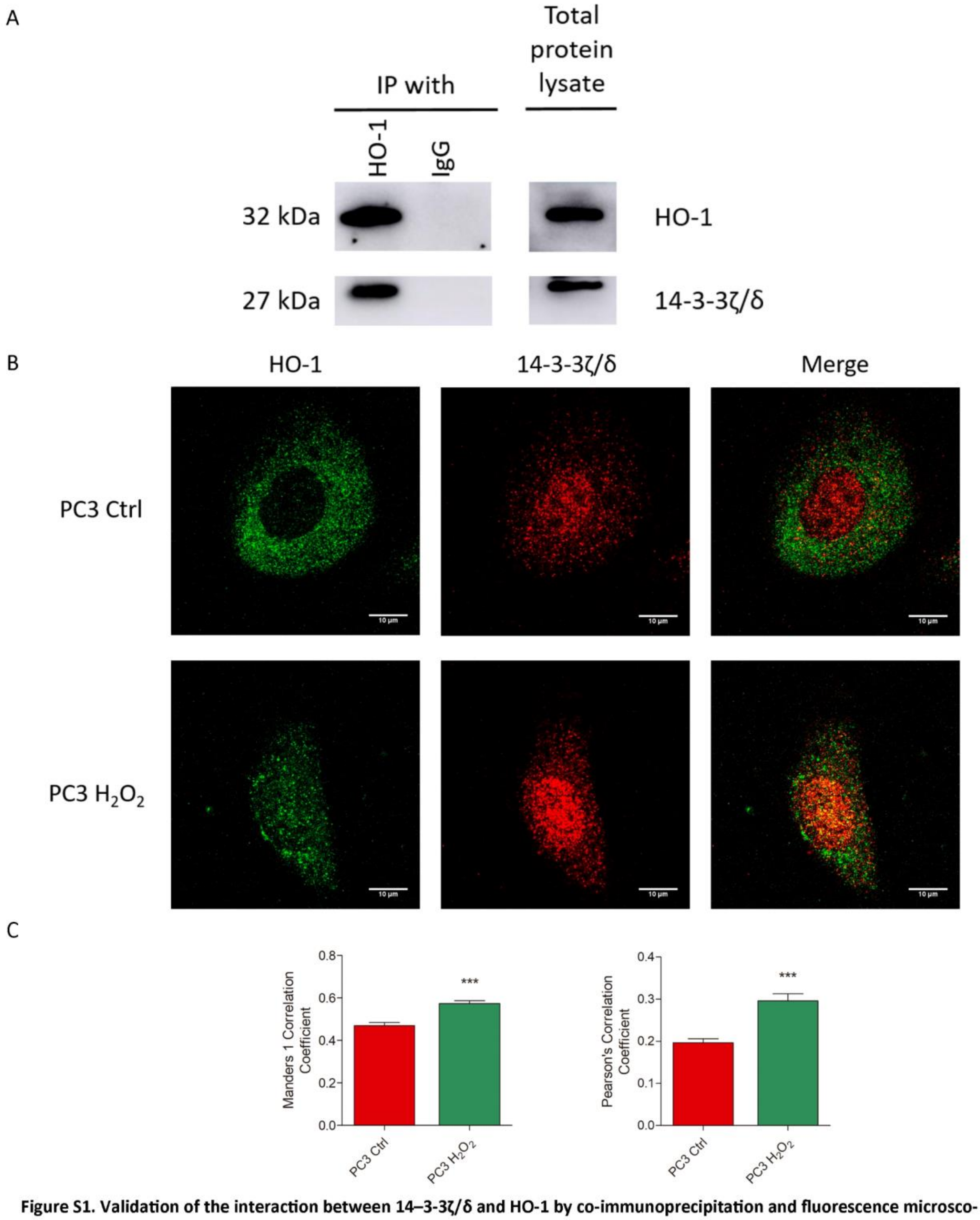

Figure S1. Validation of the interaction between 14–3-3ζ/δ and HO-1 by co-immunoprecipitation and fluorescence microscopy. A) PC3 cells were treated with H_2O_2 (200 µM, 30 min). After 24 h of treatment, cells were lysed and the lysates were subjected to immunoprecipitation (IP) with monoclonal rabbit anti-human HO-1 or anti-human IgG antibodies (control) using agarose beads. Immunoprecipitates were analyzed by Western Blot using monoclonal mouse anti-human HO-1 or rabbit anti-human 14-3-3ζ/δ antibodies. B) PC3 cells were treated or not with H_2O_2 (200 µM, 30 min). 24 h after treatment, immunofluorescence staining was performed to observe the cellular localization of endogenous HO-1 and 14-3-3ζ/δ. Scale bars denote 10 µm. More than 20 cells were analyzed for the IF assay for each condition. A representative image from each group is shown. One representative image from at least three independent experiments is shown. C) Manders (left panel) and Pearson (right panel) co-localization coefficients for PC3 cells treated with H_2O_2 (200 µM, 1 h) or control. Manders 1 = Fraction of HO-1 overlapping 14-3-3ζ/δ. Bars represent mean ± s.e.m from 3 biologic independent experiments. *$p < 0.05$; **$p < 0.01$; ***$p < 0.001$.

Ross-Adams patients' characteristics at baseline

	#	Freq. (%)
**Diagnosis Age (41-73, mean: 60.1 years)**		
< 50	6	2.91
50-59	37	17.96
60-69	61	29.61
70-79	7	3.40
>79	0	0.00
N.A.	105	50.97
**Clinical Stage**		
T1	102	49.51
T2	72	34.95
T3	25	12.14
N.A.	7	3.40
**Gleason Score**		
5	2	0.97
6	35	16.99
7 (3+4)	102	49.51
7 (4+3)	40	19.42
8	13	6.31
9	10	4.85
10	1	0.49
N.A.	3	1.46
**PSA at Diagnosis**		
< 4	11	5.34
4 - 10	134	65.05
> 10	60	29.13
N.A.	1	0.49

Table S1. Ross-Adams patients' characteristics at baseline (start of the follow-up survival analyses). Table contains age, clinical stage, Gleason score and PSA at diagnosis data.

Protein Name	Gene Symbol	Theoretical molecular mass (Da)	PSM	Unique peptides	Score	Coverage
High mobility group protein HMG-I/HMG-Y isoform a	AHCTF1	11.669	2	1	128	28%
Alpha-2-HS-glycoprotein	AHSG	40.098	1	1	47	1%
s100 calcium-binding protein A6	CACYBP	10.23	1	1	49	41%
Chromobox homolog 1	CBX1	21.519	4	2	155	11%
Chromobox homolog 3	CBX3	20.997	4	2	174	15%
Damage-specific DNA binding protein 1, 127kDa	DDB1	128.086	3	1	58	1%
Eukaryotic translation elongation factor 2	EEF2	96.246	1	1	55	2%
Filamin B	FLNB	280.185	1	1	61	1%
FtsJ3 protein	FTSJ3	84.015	1	1	53	2%
Gelsolin	GSN	31.052	4	3	203	22%
High mobility group AT-hook 1	HMGA1	34.635	3	2	51	28%
Heme oxygenase 1	HMOX1	32.798	35	5	167	25%
Heterogeneous nuclear ribonucleoprotein A2/B1	HNRNPA2B1	36.041	7	4	197	20%
Heterogeneous nuclear ribonucleoprotein D	HNRNPD	36.086	1	1	71	4%
Heterogeneous nuclear ribonucleoprotein U-like 2	HNRNPUL2	85.622	1	1	56	4%
Heterogeneous nuclear ribonucleoprotein D-like	HNRPDL	46.58	1	1	54	1%
Heat shock 27kDa protein	HSPB1	22.427	5	3	178	32%
KH-type splicing regulatory protein	KHSRP	10.362	1	1	66	20%
Kinesin-like protein KIF23	KIF23	98.842	2	1	59	1%
Matrin 3	MATR3	95.078	4	2	125	5%
Nucleosome assembly protein 1-like 1	NAP1L1	45.631	2	1	73	4%
Nucleophosmin 1	NPM1	31.09	4	2	165	12%
Plectin 1	PLEC	516.4	59	40	2406	11%
Peroxiredoxin 6	PRDX6	25011	2	1	59	9%
Purine-rich element binding protein A	PURA	35,003	1	1	66	8%
Retinoic acid induced 14	RAI14	110.617	2	1	60	1%
Regulator of chromosome condensation	RCC1	44,485	2	1	79	3%
Replication protein A 70 kDa DNA-binding subunit	RPA1	68.723	1	1	66	9%
Ribosomal protein SA pseudogene 9	RPSA	32.947	1	1	68	9%
Signal-induced proliferation-associated 1-like protein 1	SIPA1L1	201.102	1	1	47	1%
Sequestosome 1	SQSTM1	48.37	1	1	54	4%
Splicing factor, arginine/serine-rich 3	SRSF3	19.546	1	1	53	1%
Testis derived transcript	TES	49.789	2	1	52	3%
Thyroid hormone receptor associated protein 3	THRAP3	108.686	1	1	53	1%
Tropomodulin 3	TMOD3	39.727	4	2	177	9%
DNA topoisomerase 1	TOP1	66,890	2	2	115	5%
Tripartite motif-containing 28	TRIM28	80.621	11	7	463	17%
Tyrosine 3-monooxygenase/tryptophan 5-monooxygenase activation protein, beta polypeptide	YWHAB	28.179	1	1	78	11%
Tyrosine 3-monooxygenase/tryptophan 5-monooxygenase activation protein, epsilon polypeptide	YWHAE	29.326	1	1	98	11%
Tyrosine 3-monooxygenase/tryptophan 5-monooxygenase activation protein, zeta polypeptide	YWHAZ	27.899	3	2	183	17%
Zinc finger CCCH-type, antiviral 1	ZC3HAV1	103.135	3	2	219	7%

Table S2. Differential proteins identified by mass spectrometry in GST-HO-1 *vs.* GST. The table includes the protein name, the gene name, the theoretical molecular mass (KDa), the number of PSMs, the number of unique peptides, the score and the coverage. PSMs = Peptide Spectrum Matches.
